# Enzymes as modular catalysts for redox half-reactions in H_2_-powered chemical synthesis: from biology to technology

**DOI:** 10.1042/BCJ20160513

**Published:** 2017-01-06

**Authors:** Holly A. Reeve, Philip A. Ash, HyunSeo Park, Ailun Huang, Michalis Posidias, Chloe Tomlinson, Oliver Lenz, Kylie A. Vincent

**Affiliations:** 1 Department of Chemistry, Inorganic Chemistry Laboratory, University of Oxford, Oxford, OX1 3QR, U.K.; 2 Department of Chemistry, Technische Universität Berlin, Berlin 10623, Germany

**Keywords:** biotechnology, hydrogen, hydrogenase, heterogeneous catalysis, NADH, redox enzymes

## Abstract

The present study considers the ways in which redox enzyme modules are coupled in living cells for linking reductive and oxidative half-reactions, and then reviews examples in which this concept can be exploited technologically in applications of coupled enzyme pairs. We discuss many examples in which enzymes are interfaced with electronically conductive particles to build up heterogeneous catalytic systems in an approach which could be termed *synthetic biochemistry*. We focus on reactions involving the H^+^/H_2_ redox couple catalysed by NiFe hydrogenase moieties in conjunction with other biocatalysed reactions to assemble systems directed towards synthesis of specialised chemicals, chemical building blocks or bio-derived fuel molecules. We review our work in which this approach is applied in designing enzyme-modified particles for H_2_-driven recycling of the nicotinamide cofactor NADH to provide a clean cofactor source for applications of NADH-dependent enzymes in chemical synthesis, presenting a combination of published and new work on these systems. We also consider related photobiocatalytic approaches for light-driven production of chemicals or H_2_ as a fuel. We emphasise the techniques available for understanding detailed catalytic properties of the enzymes responsible for individual redox half-reactions, and the importance of a fundamental understanding of the enzyme characteristics in enabling effective applications of redox biocatalysis.

## Introduction

Metalloenzyme moieties are combined, adapted and reused in Nature to build up the modular redox circuits that provide metabolic flexibility in microorganisms. We focus on the H^+^/H_2_ cycling catalysed by hydrogenases and its combination with other biocatalysed redox half-reactions. Certain microorganisms are capable of using H_2_ as a cellular energy source or of providing reducing equivalents to support a range of cellular redox processes, whereas others produce H_2_ during fermentation [1]. Several types of hydrogenases have emerged during evolution, and here we are concerned with NiFe hydrogenase modules that incorporate a bimetallic nickel–iron active site [2]. The redox half-reaction is shown in eqn (1), together with the reduction potential calculated for pH 7, 25°C, 1 bar H_2_, but this potential shifts with H_2_ partial pressure, pH and temperature according to the Nernst equation.

1 $$2H^{+}+2e^{-}\to H_{2}\;E^{o\prime }=-0.413\;V.$$

**Figure 1.**
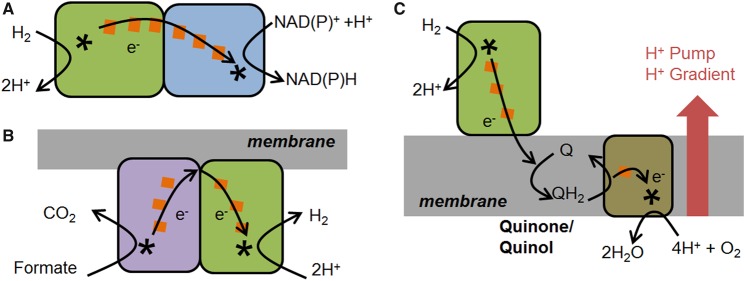
Schematic representation of some of the biological functions of NiFe hydrogenases. (**A**) H_2_ oxidation coupled to NAD(P)H generation by an NAD^+^-reducing soluble hydrogenase. (**B**) Formate oxidation coupled to H_2_ generation by part of the formate hydrogen lyase complex. (**C**) H_2_ oxidation by a membrane-bound hydrogenase coupled [via the quinone pool, Q/QH_2_ (quinone/quinol)] to O_2_ reduction and proton pumping across the inner membrane. Electron transfer relays are represented by orange squares (the number of squares does not necessarily reflect the number of naturally occurring redox centres), while catalytic sites are represented by asterisks, throughout.

There are many examples of microbial metabolic processes that incorporate the H^+^/H_2_ couple catalysed by NiFe hydrogenase moieties. Soluble, NAD(P)^+^ [nicotinamide adenine dinucleotide (phosphate) oxidised]-reducing hydrogenases that link a NiFe hydrogenase module to a flavin-containing NAD(P)^+^-reducing module are represented schematically in Figure 1A [3], and use H_2_ to generate NAD(P)H [nicotinamide adenine dinucleotide (phosphate) reduced] for CO_2_ fixation and biosynthesis of complex molecules. Formate hydrogen lyase (Figure 1B) links the reduction of H^+^ to generate H_2_ to the oxidation of formate to CO_2_ [4,5]. The NiFe hydrogenase module is found again in multicomponent systems that link periplasmic H_2_ oxidation to cytoplasmic O_2_ reduction and transmembrane proton pumping (Figure 1C) [2] to the reduction of other substrates such as fumarate or nitrate.

**Figure 2.**
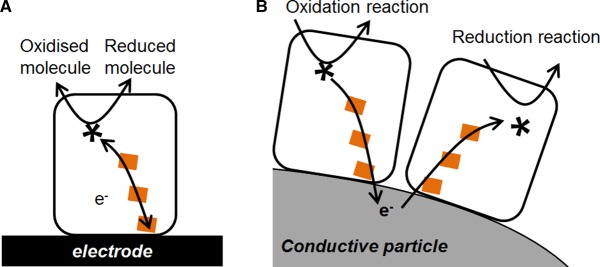
Redox enzymes containing a relay chain of electron transfer centres are well suited to exchanging electrons directly with an electronically conductive surface. This allows these enzymes to be (**A**) studied on electrodes and (**B**) coupled artificially on electronically conductive particles.

The enzyme modules that are the subject of this review transfer electrons from a redox half-reaction catalysed at a buried active site (asterisks in Figure 1) to the protein surface via a relay chain of redox centres (orange squares in Figure 1) — usually iron–sulphur clusters or haem sites. This makes them ideal subjects for investigation by direct electrochemical methods, where the protein is immobilised on an electrode and electrons are exchanged via the relay chain between the electrode and the buried catalytic site (Figure 2A). This has given rise to the widely used approach known as protein film electrochemistry [6,7]. These redox enzymes rely on direct electron transfer steps and therefore contrast with the much larger subgroup of oxidoreductases that rely on biological hydride carriers such as NADH and catalyse hydrogenation and dehydrogenation reactions by hydride transfer. In some cases, the relevant enzyme moieties are isolated soluble units, whereas in other cases they are part of large electron-transferring complexes that may be associated with biological membranes. Using genetic engineering strategies, the enzyme modules of interest may be artificially dissociated from such complexes.

In this review, we discuss how Nature's modular approach to redox catalysis can be extended further in a synthetic biochemistry approach in which enzyme redox pairs are electronically interlinked on conductive particles or surfaces. In particular, we focus on the use of H_2_ gas as a clean source of electrons (energy) to drive enzyme-catalysed reductive half-reactions for the synthesis of chemical building blocks, specialised fine chemicals and bio-derived fuels. We show how the information gained from the electrochemical study of redox enzymes can be used to inform biotechnological applications in which enzyme moieties are assembled and linked on conductive surfaces (Figure 2B).

## Biological half-reactions studied by protein film electrochemistry

Protein film electrochemistry has emerged as an effective way to control, probe and exploit catalysis of redox half-reactions by enzymes. In this approach, enzyme molecules are immobilised on an electrode, such that they engage in direct electron exchange with the electrode. In the simplest procedures, the enzyme can be directly adsorbed onto a graphite (or other carbon material) electrode; in other cases, carbon or metal electrodes are modified with appropriate chemical monolayers to aid enzyme attachment [8,9]. The electrode effectively replaces physiological electron donors or acceptors, or the artificial redox agents used in solution assays, and the current measured at the electrode reflects the flow of electrons to/from the enzyme as it engages in catalysis of reduction or oxidation reactions. Rapid rotation of the electrode can provide efficient mass transport of substrate from a large volume of bulk solution to the small quantity of immobilised enzyme, as well as removing products, so protein film electrochemistry experiments can be carried out with negligible change in the substrate concentration experienced by the enzyme. This contrasts with conventional solution assays in which substrate becomes depleted in the bulk solution as the product builds up. The potential (voltage), which is set at the electrode, controls the thermodynamic driving force (redox conditions) experienced by the immobilised enzyme. One useful type of electrochemical experiment for investigating enzyme activity is cyclic voltammetry, in which the potential is swept back and forth between two potential limits to observe changes in enzyme activity with potential. For a redox enzyme that functions reversibly, catalysis in either the oxidative or reductive direction can be initiated by taking the electrode potential to more positive or more negative values, respectively.

**Figure 3.**
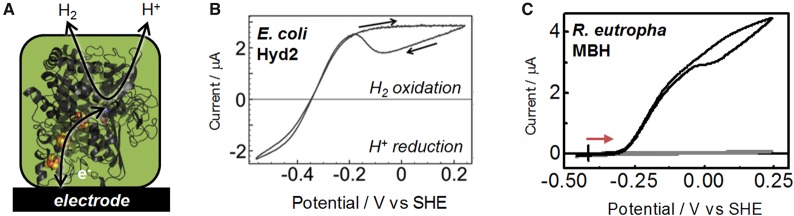
Protein film electrochemistry has been insightful in studies of the H^+^/H_2_ redox half-reaction catalysed by hydrogenases. (**A**) Schematic representation of a NiFe hydrogenase on an electrode. Cyclic voltammograms at a pyrolytic graphite ‘edge’ rotating disc electrode for: (**B**) *E. coli* hydrogenase 2 (Hyd 2, adapted with permission from Flanagan and Parkin [12]), recorded at pH 6, 3% H_2_ and (**C**) *R. eutropha* MBH (black line), overlaid on the response for a bare electrode (grey line). Conditions for (**C**): pH 7.0, 0.1 M phosphate buffer, 100% H_2_ with electrode rotation of 2400 rpm. The overpotential requirement of the enzyme is indicated by a red arrow, relative to the thermodynamic potential under these conditions (indicated by a vertical black line).

These points are well established for NiFe hydrogenases, which have been studied extensively using electrochemical approaches at graphite electrodes, as represented schematically in Figure 3A [10,11]. Carbon materials are useful as electrodes for this work because they do not catalyse background H_2_ oxidation or H^+^ reduction in the potential range needed for studying the enzymes. NiFe hydrogenases have a relay chain of iron–sulphur clusters, and in the electrochemical experiment, these allow rapid electron transfer between the electrode and the NiFe catalytic site. The reversible response of *Escherichia coli* hydrogenase 2 (Hyd2) operating under 3% H_2_ in N_2_ is shown in Figure 3B [12]. At the most negative potentials, the enzyme is taking up electrons from the electrode (giving a negative current) to reduce protons from solution to make H_2_. At more positive potentials, the enzyme works in reverse, stripping electrons from H_2_ to generate H^+^ and a positive current at the electrode. As the potential is raised further, this enzyme undergoes an oxidative inactivation reaction which is reversed as the electrode is swept back to more negative potentials on the reverse scan. Importantly, the enzyme operates with no detectable overpotential requirement relative to *E*(H^+^/H_2_) at pH 6.0, 3% H_2_ in N_2_, shown by the fact that the current *crosses* the zero line sharply. Figure 3C shows an example of a NiFe hydrogenase that is very poor at H^+^ reduction under a H_2_ atmosphere, and is strongly biased towards H_2_ oxidation, the membrane-bound hydrogenase (MBH) from *Ralstonia eutropha*. This is likely to be related to the fact that H_2_ oxidation in this enzyme requires a slight overpotential relative to *E*(H^+^/H_2_): the onset of H_2_ oxidation is ∼0.1 V more positive than *E*(H^+^/H_2_) calculated for the conditions of this experiment. Separate protein film electrochemistry experiments on *R. eutropha* MBH have revealed that it is sufficiently O_2_-tolerant that it can continue oxidising H_2_ in air [13,14].

More than 10 different isolated NiFe hydrogenases have been studied on carbon electrodes, revealing a range of catalytic properties [8,15–17]. For example, the NiFe hydrogenase I from the hyperthermophile *Aquifex aeolicus* is both O_2_-tolerant and stable at high temperatures [17], but like *R. eutropha* MBH is afflicted with a small overpotential requirement [15]. The robust hydrogenase I from *E. coli* (Hyd1) is O_2_-tolerant and also operates with a slight overpotential requirement, whereas the O_2_-sensitive Hyd2 from *E. coli* operates at no notable overpotential (Figure 3B) but is sensitive to O_2_ [18]. The properties of hydrogenases as electrocatalysts have been altered further by site-directed amino acid exchanges [19,20]. Importantly, many NiFe hydrogenases are extremely active for H_2_ oxidation with electrocatalytic turnover frequencies often estimated to exceed 1000 s^−1^ [21,22]. The related NiFeSe hydrogenases, in which one of the cysteinyl sulphur atoms co-ordinating the active site is replaced by a selenium, are also promising candidates for applications, showing high activity and lower sensitivity to O_2_ than some of the NiFe hydrogenases [23]. The set of characterised NiFe(Se) hydrogenases provides a useful library of bio(electro)catalysts for the H_2_ oxidation half-reaction under different conditions for the applications discussed below. Many FeFe hydrogenases have also been characterised electrochemically and many are highly active electrocatalysts, particularly in the H_2_ production direction, but they tend to be more difficult to handle owing to their greater sensitivity to O_2_ [24–26].

**Figure 4.**
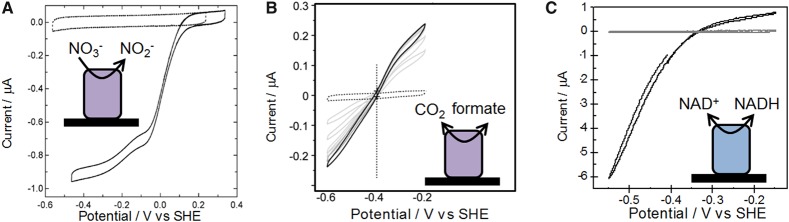
Protein film electrochemistry used to probe enzyme-catalysed redox half-reactions. (**A**) Work from Elliott et al. demonstrating nitrate reduction by the NarGHI subcomplex of *E. coli* nitrate reductase in the presence of 1 mM $NO_{3}^{-}$ at pH 7, 30°C, adapted with permission from ref. [27]. Copyright 2014 American Chemical Society. (**B**) Data from Bassegoda et al. showing reversible interconversion of CO_2_ and formate by *E. coli* Mo-containing formate dehydrogenase in the presence of 1 mM CO_2_ and 1 mM formate at pH 6.8, 23°C (dark scan recorded first; subsequent scans showing evidence of enzyme desorption are shown in grey) adapted with permission from ref. [28]. (**C**) Work from Lauterbach et al. showing reversible NAD^+^/NADH cycling by the NAD^+^-reducing HoxFU moiety of *R. eutropha* soluble hydrogenase in the presence of 1 mM NAD^+^ and 1 mM NADH at pH 8, 30°C, adapted with permission from ref. [29].

Protein film electrochemistry has been equally instructive in studies of other enzyme-catalysed redox half-reactions. A selection of examples is shown in Figure 4. Figure 4A shows a cyclic voltammogram revealing nitrate reduction to nitrite by the NarGHI subcomplex of *E. coli* nitrate reductase [27]. Figure 4B shows reversible cycling of formate and CO_2_ by a Mo-containing formate dehydrogenase moiety, a reaction which holds promise for fixation of CO_2_ to generate fuels or useful chemical building blocks [28]. Figure 4C shows NAD^+^ reduction/NADH oxidation by the NAD^+^-reducing moiety of *R. eutropha* soluble hydrogenase (blue in Figure 1A), which has been separated from the hydrogenase moiety by protein engineering [29]. The ability of certain immobilised enzyme moieties to carry out the NAD^+^/NADH reaction with no detectable overpotential [29,30] is particularly significant from a catalytic perspective since electrocatalytic NADH oxidation or NAD^+^ reduction is difficult to achieve with standard electrodes without large overpotential requirements [31,32].

Other examples of redox enzymes functioning well in direct electron transfer at electrodes include carbon monoxide dehydrogenase (CODH) for reversible interconversion of CO and CO_2_ [33], fumarate reductase for interconversion of fumarate and succinate [34], cellobiose dehydrogenase for oxidation of substrates such as lactate [35], fructose dehydrogenase for oxidation of fructose [36,37] and laccase or bilirubin oxidase for O_2_ reduction [38–40].

**Figure 5.**
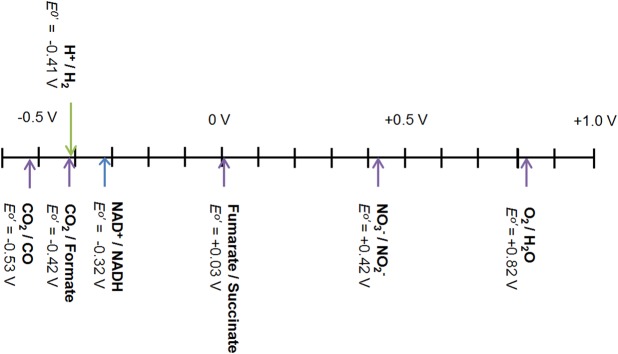
The thermodynamic context for coupling biological half reactions. Thermodynamic potentials for relevant biological couples at pH 7.

The potentials of a range of biologically relevant redox half-reactions are shown in Figure 5. The potentials of the H^+^/H_2_ and O_2_/H_2_O couples are −0.413 and +0.816 V under standard conditions but corrected for pH 7. This large and favourable gap in potential (Δ*E* = 1.23 V) is exploited by organisms, such as *R. eutropha*, for energy generation from H_2_ as shown schematically in Figure 1C. With this significant energetic driving force, a moderate overpotential requirement in the enzymes for H_2_ oxidation or O_2_ reduction is not problematic. However, other biological systems rely on linking redox half-reactions which are much more closely spaced in potential. An example is the soluble hydrogenase shown in Figure 1A, which couples H_2_ oxidation to NAD^+^ reduction. The Δ*E* for the H^+^/H_2_ and NAD^+^/NADH redox couples at pH 7.0 is just 0.11 V; therefore, any overpotential requirement for either NAD^+^ reduction or H_2_ oxidation would significantly affect the success of coupling these two half-reactions. In accordance with this, both the hydrogenase moiety and the NAD^+^-reductase moiety (Figure 4C) of this enzyme have been shown to work reversibly without detectable overpotential requirement [29,41].

The following sections discuss methods for artificially coupling enzyme-catalysed half-reactions, with an emphasis on application to H_2_-driven chemical synthesis.

## Coupled redox catalysis on carbon particles

**Figure 6.**
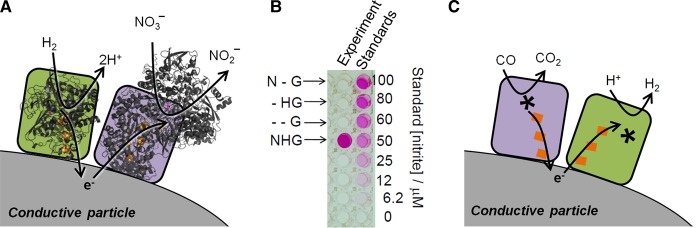
The concept of coupled enzyme redox catalysis on electronically conductive graphite platelet particles. (**A**) Schematic representation of hydrogenase and nitrate reductase co-immobilised on graphite particles. (**B**) Results from a Griess reagent assay showing that H_2_-driven reduction of nitrate to nitrite by enzyme-modified particles (NHG) occurs only when nitrate reductase (N) and hydrogenase (H) are co-adsorbed on the graphite particles (G), but not in the absence (−) of any of these components. Panel (**B**) is reproduced from work by Vincent et al. with permission from ref. [42]. (**C**) Schematic representation of hydrogenase and carbon monoxide dehydrogenase co-immobilised on carbon particles for catalysis of the water-gas shift reaction.

The observation that enzyme-modified graphite electrodes are efficient electrocatalysts for a wide range of redox half-reactions inspired Armstrong and co-workers to bring together two enzyme-catalysed half-reactions on graphite platelet particles. The graphite particle serves as a sink for electrons from the oxidation half-reaction, and a donor to the enzyme catalysing a reduction reaction, and thus can be considered as a ‘wire’ for connecting the two redox enzyme moieties (Figure 2B). This was demonstrated for particles modified with both a NiFe hydrogenase (from *Allochromatium vinosum*) and the *E. coli* nitrate reductase moiety from Figure 4A, which are shown schematically in Figure 6A [42]. Nitrite was detected using the colorimetric Griess reagent assay when particles modified with these two enzymes were immersed in a buffered solution containing sodium nitrate and flushed with H_2_ (Figure 6B). No nitrite was detected in the absence of particles, confirming that electron transfer must occur via the graphite. A second example from this proof-of-concept study showed that fumarate reduction to succinate by fumarate reductase (*E. coli* FrdAB) can be coupled to H2 oxidation by a co-immobilised hydrogenase. Although nitrate and fumarate reduction reactions are not obviously important in chemical synthesis, this work demonstrates a powerful modular concept for artificially coupling selective biocatalysis of redox half-reactions [42]. The authors estimated an enzyme loading of ∼1600 enzyme molecules per platelet particle [42], and presumably, the hydrogenase and reductase enzyme will be randomly mixed on the surface.

Armstrong and co-workers went on to exploit graphite platelets modified with hydrogenase and CODH as a heterogeneous catalyst for the water–gas shift reaction, in which CO and water combine to release H_2_ and CO_2_; here, the hydrogenase operates in reverse to reduce H^+^ to H_2_ (Figure 6C) [43].

## Modular H_2_-driven NADH generation

**Figure 7.**
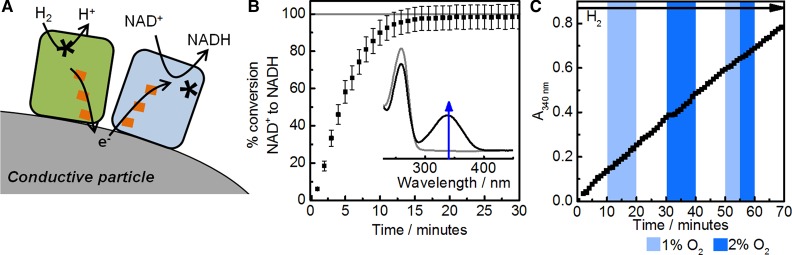
Oxidation of H_2_ coupled to NAD^+^ reduction on carbon particles to yield NADH. (**A**) Schematic representation of enzyme-modified particles for H_2_-driven NADH generation. (**B**) Conversion of NAD^+^ to NADH by carbon black [Black Pearls (BP) 2000] particles modified with *E. coli* Hyd2 [18] and the NAD^+^-reducing moiety (HoxH^I64A^YFU) of *R. eutropha* soluble hydrogenase with the hydrogenase portion inactivated by an I64A exchange in the hydrogenase large subunit (prepared according to established protocols) [46] operating in a H_2_-saturated solution containing NAD^+^ (70 µM) at pH 8.0. Figure reproduced with permission from ref. [45] by Reeve et al. (**C**) H_2_-driven NADH generation using enzyme-modified particles in the presence of H_2_:O_2_ mixtures. This experiment used *E. coli* Hyd1 and the same NAD^+^-reducing enzyme moiety immobilised on carbon black in a starting solution of NAD^+^ (1 mM); the gas mixture in the experiment headspace was precisely controlled and changed throughout the experiment using mass flow controllers; H_2_ was present throughout.

We have demonstrated a related approach for coupling H_2_ oxidation to NADH generation on carbon particles, shown schematically in Figure 7A [44,45]. The observation that the NAD^+^-reducing moiety of *R. eutropha* soluble hydrogenase on a graphite electrode is an efficient electrocatalyst for NAD^+^ reduction and NADH oxidation (see Figure 4C) inspired us to combine this NAD^+^-reductase with a robust hydrogenase on carbon surfaces to assemble an alternative heterogeneous catalyst system for NAD^+^ reduction to NADH. Near-complete conversion of NAD^+^ to NADH can be achieved using this system with a hydrogenase that operates close to the thermodynamic potential for H_2_ oxidation (i.e. with no overpotential requirement) such as *E. coli* Hyd2 or *Desulfovibrio vulgaris* Miyazaki F hydrogenase. Figure 7B shows the percentage of conversion of NAD^+^ to NADH during a reaction course under a H_2_ atmosphere measured by the increase in absorbance at 340 nm as the cofactor becomes reduced. These catalytic enzyme-modified particles were shown to operate at H_2_ partial pressures as low as 0.02 bar, and can be separated from the reaction solution and reused over multiple reaction cycles [45]. No NADH is detected from a reaction mixture involving the two enzymes but no carbon particles, showing again that the carbon is essential in providing electronic coupling between the enzymes.

Having enzymes ‘plugged in’ to conductive particles allows a very modular approach to coupling redox half-reactions. Drawing on the library of available isolated NiFe hydrogenases with different properties, we then selected an O_2_-tolerant hydrogenase, *E. coli* Hyd1, to demonstrate NADH generation under H_2_ in the presence of 1–2% O_2_. As shown in Figure 7C, this level of O_2_ does not have a significant effect on the rate of NADH generation, as indicated by the fairly linear slope for change in absorbance at 340 nm with time.

**Figure 8.**
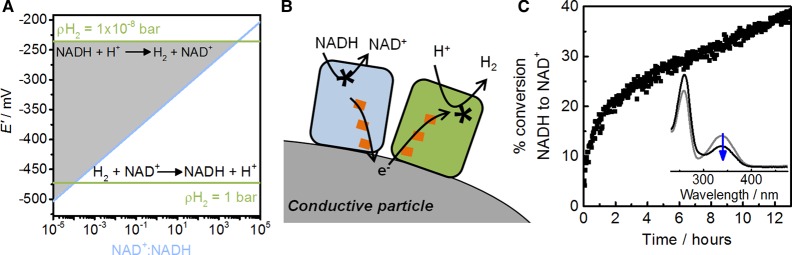
Oxidation of NADH to NAD^+^ coupled to H_2_ generation on carbon particles. (**A**) Thermodynamics for H_2_ + NAD^+^ ↔ H^+^ + NADH at high and low H_2_ partial pressures (*p*H_2_) and a range of NAD^+^:NADH ratios. (**B**) Schematic representation of enzyme-modified particles for NADH oxidation and H_2_ production. (**C**) Conversion of NADH to NAD^+^ using H^+^ reduction as the electron sink at carbon black particles modified with *E. coli* Hyd2 [18] and the NAD^+^-reducing HoxH^I64A^YFU of *R. eutropha* in the presence of NADH (1 mM) at pH 8, 30°C.

The reduction potentials for the H^+^/H_2_ and NAD^+^/NADH couples are close together (see Figure 5), meaning that subtle changes in solution conditions can reverse the thermodynamically favourable direction of reaction. This is illustrated in Figure 8A, which shows how the H^+^/H_2_ couple potential varies with H_2_ partial pressure (*p*H_2_) and the NAD^+^/NADH couple potential varies with the ratio of NAD^+^:NADH. At high levels of H_2_ and a high ratio of NAD^+^/NADH (lower portion of Figure 8A), the reduction of NAD^+^ by H_2_ is favoured. However, when both the H_2_ partial pressure and the NAD^+^/NADH ratio are low (upper shaded portion of Figure 8A), the reaction will occur in reverse. Enzyme-modified carbon particles operating in the H^+^ reduction direction are shown schematically in Figure 8B. For this work, two enzymes that clearly exhibit bidirectional behaviour in electrochemical experiments were chosen: Hyd2 from *E. coli* (see Figure 3B) and the NAD^+^-reducing moiety from *R. eutropha* soluble hydrogenase (see Figure 4C). Results for NAD^+^ generation from a solution containing NADH under a N_2_ atmosphere are shown in Figure 8C.

**Figure 9.**
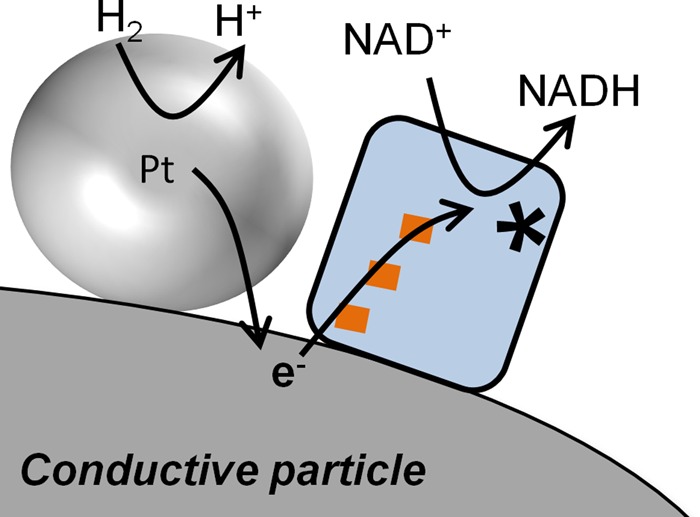
A composite chemo-/bio- approach to heterogeneous catalysis of H2-driven NADH generation. This approach involves co-immobilised Pt nanoparticles for H2 oxidation and an NAD^+−^ reducing enzyme moiety for NAD^+^ reduction.

There has also been interest in metal-catalysed or combined metal/enzyme systems for H_2_-driven NADH recycling. Wang and Yiu [47] tested Pt on an Al_2_O_3_ support for H_2_-driven NADH generation. Depending on the reaction conditions, various incorrect forms of the cofactor were formed using this catalyst, but some successful NADH recycling was achieved. Reeve et al. [44] explored a composite catalyst, shown schematically in Figure 9 comprising electrochemically platinised pyrolytic graphite platelets with immobilised NAD^+^-reducing enzyme moiety (HoxFU from *R. eutropha*), or NAD^+^-reducing HoxH^I64A^YFU (I64A exchange in the subunit, HoxH) of *R. eutropha* immobilised on HiSPEC 3000 carbon black powder with 20% Pt loading. In both cases, the presence of the NAD^+^-reducing enzyme gave rise to a significantly faster rate of NADH generation compared with the respective carbon-supported platinum catalyst alone. For the NAD^+^-reducing enzyme on HiSPEC 3000 carbon black with 20% Pt, 92% conversion of NAD^+^ to NADH was achieved, although possible impurities in the NADH due to the presence of the Pt were not analysed. Besides concerns over formation of bio-inactive cofactor derivatives in the presence of precious metals, these systems have the disadvantage of requiring an expensive and potentially toxic metal catalyst.

The examples presented so far demonstrate possibilities for turning redox enzyme pairs into selective heterogeneous catalysts which can be handled easily, and separated from solution and reused. In the following section, we present experiments that explore further the flow of electrons between redox enzymes linked on carbon particles, focusing again on hydrogenase paired with an NAD^+^-reducing moiety.

## Probing the electronic communication between co-immobilised hydrogenase and NAD^+^-reductase

**Figure 10.**
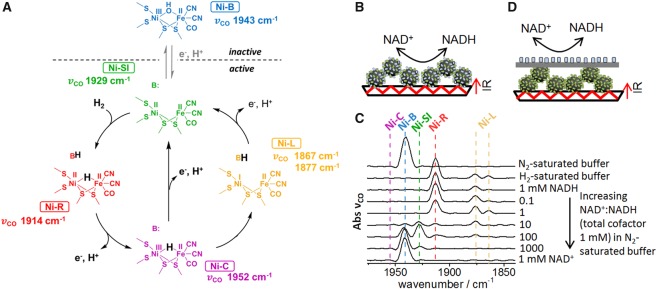
Attenuated ATR IR spectroscopy used to probe the electronic connection between the hydrogenase and NAD^+^-reducing enzyme moiety on carbon particles. (**A**) Main inactive and active states of the catalytic site of *E. coli* Hyd1, together with the *v*_CO_ wavenumber positions observed for each state. B: indicates basic site(s) that accept protons during catalysis, which could include co-ordinated cysteine sulphur atoms, and other surrounding amino acid residues. (**B**) Schematic representation of the experimental set-up for testing particles modified with a mixture of hydrogenase and NAD^+^-reducing moiety. (**C**) Set of ATR IR spectra recorded during solution exchange experiments. The cell solution contained 50 mM Tris–HCl (pH 8.0), and the H_2_/N_2_ and NAD^+^/NADH concentrations were varied as indicated on the figure. (**D**) Schematic representation of the experimental set-up for testing the immobilised enzymes physically separated by a piece of carbon paper (Toray TGP-H-030), in response to changes in solution H_2_/N_2_ or NAD^+^/NADH ratio. For these experiments, the hydrogenase was *E. coli* Hyd1 [18], and the NAD^+^-reducing moiety was HoxH^I64A^YFU of *R. eutropha* soluble hydrogenase, with the hydrogenase portion inactivated by an I64A exchange in the HoxH subunit (prepared according to established protocols) [46]. Carbon particles were BP2000 carbon black.

NiFe hydrogenases are fairly well characterised spectroscopically, and in particular, infrared (IR) spectroscopy has been useful in understanding states of the active site. The Fe atom of the active site of NiFe hydrogenases has native CO and CN^−^ ligands which give rise to clearly distinguishable absorption bands in the IR spectrum, and the wavenumber positions of these bands vary according to the redox level of the active site. IR experiments have been performed under electrochemical control on many NiFe hydrogenases, so the redox levels of the active site present at different applied potentials are well documented [48–50]. The wavenumber positions of the CO stretching vibrational bands, *v*_CO_, for some of the inactive and active states of the catalytic site of *E. coli* Hyd1 are indicated in Figure 10A. Here, we make use of this information as a guide to follow the redox state of hydrogenase (*E. coli* Hyd1) in response to coupled catalysis with an NAD^+^-reducing moiety from *R. eutropha*.

To confirm that the hydrogenase responds to electron transfer from the coupled NAD^+^-reducing enzyme moiety, we initially carried out experiments with carbon particles modified with a mixture of the hydrogenase and the NAD^+^-reducing moiety in an attenuated total reflectance (ATR) IR mode, as shown schematically in Figure 10B. Particles were deposited on a silicon internal reflection element, such that the ATR IR cell solution could be exchanged easily [49]. This allows direct comparison of the coupled enzyme system under a range of conditions, as shown by the spectra in Figure 10C. Hyd1 seems to be isolated mainly in the catalytically inactive state known as Ni-B, and this is reflected in the spectrum of the as-prepared particles (top spectrum, labelled ‘N-saturated buffer’). The hydrogenase was then activated using H_2_ (labelled ‘H-saturated buffer’), giving rise to peaks corresponding to reduced, catalytically active states of the active site, predominantly Ni-R and Ni-L. The solution was then exchanged for N_2_-saturated solution containing 1 mM NADH (labelled ‘1 mM NADH’). Introduction of the reduced cofactor had no further effect on the hydrogenase redox level. The solution was then exchanged for a series of N_2_-saturated solutions containing increasingly higher ratios of NAD^+^/NADH. Raising the proportion of the oxidised cofactor NAD^+^ in solution gave rise to increasingly oxidised levels of the hydrogenase active site (enriched in Ni-SI and Ni-B), until at 1 mM NAD^+^ (labelled ‘1 mM NAD^+^’) when the hydrogenase reverted entirely to the oxidised inactive state, Ni-B. Clearly, making the solution NAD^+^/NADH pool increasingly oxidised by raising the proportion of NAD^+^ leads to electron flow out of the hydrogenase towards the NAD^+^-reducing enzyme moiety, causing the hydrogenase to become increasingly oxidised. In a control experiment in which hydrogenase-modified particles were set up in the ATR IR cell in the absence of NAD^+^-reducing enzyme moiety, no changes were observed in the *v*_CO_ bands of the hydrogenase active site in response to variation in NAD^+^ or NADH in solution.

To translate biocatalytic systems into viable technologies, it is essential to fully understand the activation, inactivation and inhibition processes occurring in the enzyme components, such that robust handling and operating protocols can be developed. The ATR IR method allows *in operando* study of an enzyme heterogeneous catalyst system, providing insights into the processes occurring under different substrate (or other) conditions. For example, the spectra in Figure 10C show that the addition of 1 mM NAD^+^ under N_2_ is sufficient to completely oxidise the hydrogenase to its catalytically inactive Ni-B state, from which reactivation with either NADH or H_2_ would be required, and thus are informative as to conditions which can lead to inactivation of the enzymes.

A separate set of experiments was conducted on particles modified with hydrogenase, physically separated by a sheet of electronically conductive carbon paper from the NAD^+^-reducing moiety, which was immobilised on a second sheet of carbon paper, as shown schematically in Figure 10D. These gave similar spectral changes in response to varying the relative concentrations of NAD^+^ and NADH, confirming that the changes in the hydrogenase redox state with NAD^+^/NADH ratio must be due to electron flow between the two enzymes via the conductive carbon, and that electron transfer does require direct interaction between the two enzymes.

In this work, only the active site of the hydrogenase was monitored; however, it is also possible to follow the redox state of the flavin site of an electrode-immobilised flavoenzyme using IR spectroscopy, and it should therefore be possible to monitor electron flow between the NiFe hydrogenase site and the flavin site of the NAD^+^-reductase simultaneously [51,52].

## Using H_2_-driven NADH recycling to support dehydrogenase catalysis for applications in fine chemical synthesis

There is growing interest in harnessing the selectivity of NADH-dependent redox enzymes *in vitro* for fine chemical synthesis particularly for hydrogenation and dehydrogenation reactions. NAD(P)H cofactors also provide the reducing equivalents necessary for reduction of O_2_ by cytochrome P450 monooxygenases, which insert an O atom into a CH bond. Although enzyme catalysts are highly attractive for these reactions in terms of their regio- and stereo-selectivity, difficulties in recycling the expensive reduced cofactors have been impediments to widespread application. One established method for regeneration of NADH uses formate dehydrogenase to couple reduction of the oxidised cofactor, NAD^+^ to the oxidation of formate, but the slow turnover frequency of this enzyme and complications of solution acidification by the product, CO_2_, make this system unattractive. Alternative cofactor recycling systems include alcohol dehydrogenase-catalysed NAD(P)^+^ reduction coupled to isopropanol oxidation to acetone, or glucose dehydrogenase-mediated NAD(P)^+^ reduction linked to the oxidation of glucose to gluconolactone. These approaches to regenerating the hydride carriers have poor atom efficiency and thus generate unwanted byproducts. This has an impact on the economic and environmental viability of such enzyme cascade reactions.

The possibility of exploiting soluble NAD(P)^+^-reducing hydrogenases for H_2_-driven recycling of reduced nicotinamide cofactors has not escaped notice due to the perfect atom economy offered by hydrogenations using H_2_ as the reductant. Payen et al., for instance, reported in 1983 that the NAD^+^-reducing soluble hydrogenase from *R. eutropha* (formerly *Alcaligenes eutrophus*) co-immobilised on corn stover particles with l-lactate dehydrogenase functions in sustained pyruvate reduction with a total turnover number (TTN) of 111 product per NADH over 45 h, although activity dropped significantly after ∼15 h [53]. An NADP^+^-reducing hydrogenase from *Pyrococcus furiosus* has been demonstrated for ketone reduction reactions [54]. Ratzka et al. [55] demonstrated an impressive TTN of 143 000 for the NAD^+^-reducing hydrogenase during NADH supply to a carbonyl reductase (alcohol dehydrogenase, ADH) for acetophenone reduction using the NAD^+^-reducing soluble hydrogenase from *R. eutropha* H16, despite limited stability of the enzyme (half-life, *t*_1/2_, ∼5 h). Stability of the soluble, NAD(P)^+^-reducing hydrogenases has remained a barrier to their exploitation in biotechnology.

**Figure 11.**
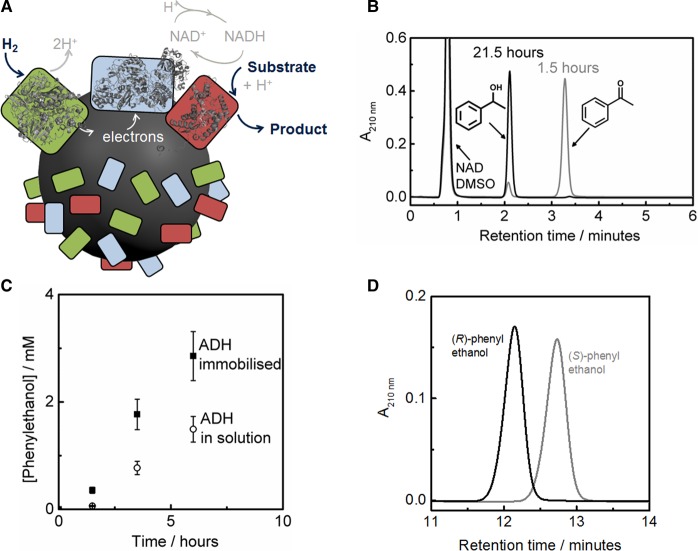
Enzyme-modified particles for highly selective H_2_-driven ketone reduction at 1 bar H_2_. (**A**) Schematic representation of the particle system. (**B**) HPLC traces after 1.5 and 21 h of H_2_-driven ketone reduction. (**C**) The generation of phenylethanol using ADH co-immobilised on particles or in solution. Panels (**B**) and (**C**) are reproduced from ref. [45] by Reeve et al. (**D**) HPLC traces showing generation of (*R*)- or (*S*)-1-phenylethanol with >99% *ee* when co-immobilising different alcohol dehydrogenases; black = (*R*)-selective ADH 101 and grey = (*S*)-selective ADH 105, both provided by Johnson Matthey Catalysis and Chiral Technologies. All experiments made use of *E. coli* Hyd2 [18] and the NAD^+^-reducing moiety was HoxH^I64A^YFU of *R. eutropha* soluble hydrogenase, with the hydrogenase portion inactivated by an I64A exchange in the HoxH subunit (prepared according to established protocols) [46] immobilised on carbon black (BP2000). Reaction conditions: 10 mM acetophenone, 1 mM NAD^+^, Tris–HCl buffer (pH 8), 25°C, 2% DMSO.

We have shown that the carbon particles modified with hydrogenase and the NAD^+^-reducing enzyme moiety described above can be extended to H_2_-driven NADH supply for NADH-dependent enzymes, as shown schematically in Figure 11A. We have reported this for H_2_-dependent reduction of pyruvate to lactate using lactate dehydrogenase [44] and acetophenone to phenylethanol using an ADH [45]. The use of H_2_ as the reducing equivalent with the heterogeneous enzyme catalyst system means that no byproducts are generated from the cofactor recycling and guarantees highly pure chemical products. Under atmospheric H_2_ pressures, >98% conversion of acetophenone to phenylethanol is achieved, as shown in Figure 11B. When using the ADH co-immobilised in close proximity to the cofactor recycling enzymes, the reaction was shown to proceed approximately twice as fast as when the ADH was used in solution under otherwise identical conditions (Figure 11C). Low cofactor concentrations (down to 10 µM) can be used; this is low enough that the cofactor does not necessarily need to be removed from the final product as it can be present at <0.1 mol%. The enzymes and the cofactor operate with high stability: each cofactor can be used at least 600 times (TN > 600) and each NAD^+^ reductase can be used more than 150 000 times (TTN > 150 000) [45]. Using either an (*R*)- or(*S*)-selective ADH (ADH 101 or 105 from Johnson Matthey Catalysis and Chiral Technologies), either enantiomer of phenylethanol can be generated with >99% enantiomeric excess (ee) as shown in the HPLC traces in Figure 11D.

## Related photocatalytic approaches

**Figure 12.**
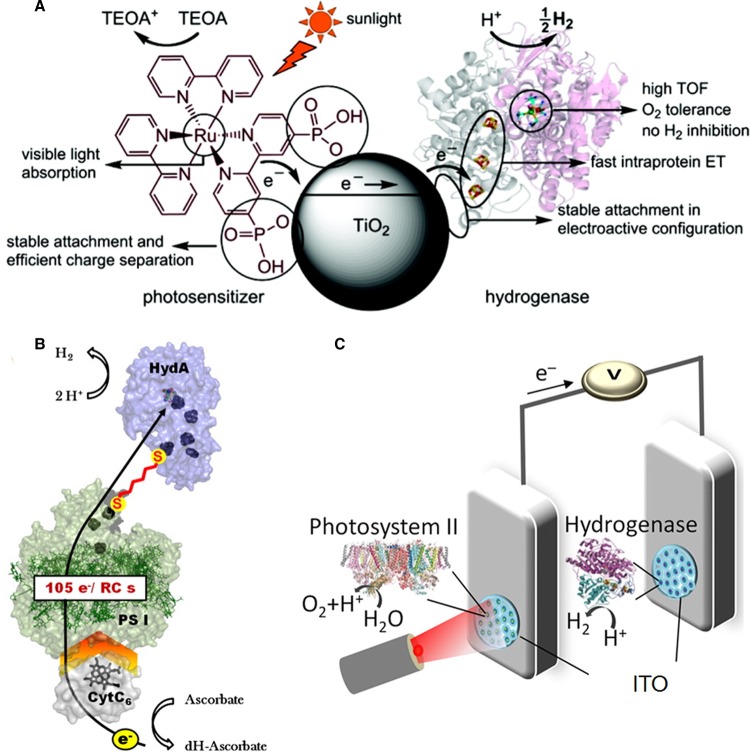
Schematic representations of selected photobiocatalytic systems. (**A**) Photocatalytic H_2_ production by *Desulfomicrobium baculatum* NiFeSe hydrogenase immobilised on TiO_2_ nanoparticles that were dye-sensitised with an Ru complex, as reported by Reisner et al. TEAO was the sacrificial reductant. This hydrogenase was selected for characteristics such as its high TOF and fast intramolecular electron transfer (ET). Reproduced with permission from ref. [56]. (**B**) Photocatalytic H_2_ production using *Synechococcus* sp. photosystem I (PS I) linked to *Clostridium acetobutylicum* FeFe hydrogenase (HydA) via a molecular dithiolate wire (red), reproduced with permission from ref. [62] by Lubner et al. Copyright 2011 National Academy of Sciences. (**C**) Photocatalytic water splitting by electronically interlinked *Thermosynechococcus elongatus* photosystem II and *D. baculatum* NiFeSe hydrogenase on separate ITO surfaces. Reproduced with permission from ref. [68] by Mersch et al.

A related body of research has demonstrated the use of redox enzymes interfaced with semiconductor or quantum dot particles for light-driven catalysis of specific reductive redox half-reactions, supported by the oxidation of a sacrificial reductant in solution such as triethanolamine (TEOA), 2-(*N*-morpholino)ethanesulfonic acid buffer or ascorbic acid (AA). Illumination excites electron transfer from sites in the particle into the enzyme, initiating reductive catalysis in the enzyme, and electron vacancies (h^+^) in the particle are refilled by donation from the sacrificial donor. This concept has been of significant interest for clean light-driven production of H_2_ as a fuel from water. This has been demonstrated with a NiFeSe hydrogenase on Ru dye-sensitised TiO_2_ particles (Figure 12A) [56], with the NiFeSe hydrogenase selected for this work, out of the library of available isolated NiFe and NiFeSe hydrogenases, because it adsorbed well on the TiO_2_, showed a high turnover frequency (TOF) for H^+^ reduction with minimal product inhibition by H_2_ and had good tolerance to O_2_. Photocatalytic H_2_ production has also been demonstrated by FeFe hydrogenases on CdTe nanocrystals [57] or CdS nanorods [58]. Again, the photocatalytic reaction concept is modular, and other biocatalysed reductive half-reactions have been demonstrated on semiconductor or quantum dot particles, including reduction of CO_2_ to CO by CODH on Ru dye-sensitised TiO_2_ particles [59,60] and reduction of N_2_ to ammonia in a system involving the nitrogenase MoFe protein on CdS nanorods [61].

**Figure 13.**
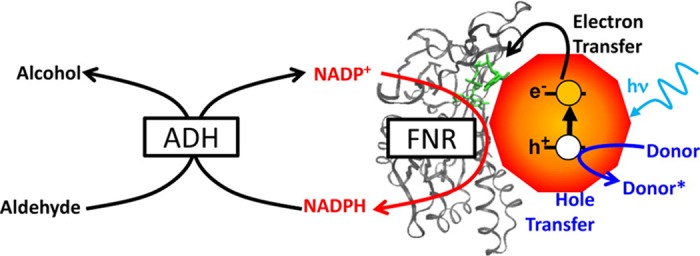
Photobiocatalytic chemical synthesis. Light-driven reduction of NADP^+^ to NADPH by ferredoxin NADP^+^-reductase on a CdSe quantum dot with AA (Donor), reduced to an ascorbyl radical (dHA, Donor*), as sacrificial reductant is used with ADH in solution for NADPH-dependent aldehyde reduction as reported by Brown et al. Reproduced with permission from ref. [70].

The desirability of clean production of H_2_ as a fuel from water has spurred the development of alternative photocatalytic approaches that link the light-harvesting photosystem I protein to either FeFe hydrogenases as shown schematically in Figure 12B [62–64], or to NiFe hydrogenases [58]. The Golbeck group have developed a strategy for linking the outer iron–sulphur clusters on hydrogenase and photosystem I via dithiolate linkers (short enough to allow electron tunnelling), which replace one of the cysteinyl ligands to each cluster as shown by the red linker in Figure 12B [62,64], whereas Lenz and co-workers have created fusion proteins of NiFe hydrogenase and an electron transfer unit of photosystem I by genetic engineering [65–67].

To avoid the requirement for the addition of sacrificial organic molecules as reductants for photobiocatalytic reductions, the ideal electron donor would be water (oxidised to O_2_), but this requires an effective catalyst for the water oxidation reaction, in addition to the light energy required to overcome the unfavourable thermodynamics (for example, Δ*E* for coupling H^+^ reduction to generate H_2_ with H_2_O oxidation to O_2_ at pH 7 is −1.23 V). An example of this concept is provided by the work of Mersch et al., represented schematically in Figure 12C, in which an indium tin oxide (ITO) surface modified with NiFeSe hydrogenase is electronically connected to another ITO surface modified with water-oxidising photosystem II [68].

Photobiocatalytic approaches have also been developed in the area of cofactor-dependent chemical synthesis. Brown et al. [70] have demonstrated a system for reduction of NADP^+^ to NADPH for supply to a NADPH-dependent ADH using a ferredoxin NADP^+^-reductase on a CdSe quantum dot with a high excess of ascorbic acid as a sacrificial electron donor (Figure 13). Although this holds promise as an alternative route to recycling reduced cofactors, the system was limited by instability of the quantum dot–enzyme complex under illumination and is also likely to suffer from the known photoinstability of the reduced cofactors [69]. As is the case with conventional NAD(P)H recycling systems involving glucose, alcohol or formate dehydrogenases that act on sacrificial organic substrates, the requirement for excess ascorbic acid as reductant in this system also compromises the atom efficiency and product purity obtained in the biocatalytic process. A further challenge in the development of quantum dot or semiconductor particle biocatalytic systems is the uncertain ‘potential’ effectively applied at the particle surface under illumination, which could lead to non-selective redox chemistry involving the cofactor or the desired reagent or product.

## Conclusion

The present paper has reviewed the biological and technological applications of redox enzymes as modular catalysts for linking selective redox half-reactions, with a focus on connecting the H^+^/H_2_ couple to other redox couples. Protein film electrochemistry is highlighted as a useful tool for understanding the individual characteristics of different redox enzymes, revealing important operational features such as catalytic bias and any overpotential requirement, which form critical background knowledge for selecting and designing enzyme pairs for artificially coupling on conductive particles. The ATR IR spectroscopic approach, demonstrated here for particles modified with hydrogenase and NAD^+^-reductase, makes it possible to characterise the redox state of hydrogenase on particles during operational catalysis. This strategy provides an understanding of the redox environment imposed by the particle on the hydrogenase under different solution conditions, and reveals conditions which lead to inactivation or damage to the enzyme. It is thus possible to assemble a large body of knowledge about the characteristics of each enzyme and their suitability for coupled catalysis that informs selection of particular enzyme modules for specific tasks. The synthetic biochemistry approach we describe here for coupling enzyme pairs on conductive particles is likely to be particularly useful in reactions involving the H^+^/H_2_ redox couple applied to H_2_-driven synthesis of fine chemicals (or the reverse reaction, production of chemicals linked to generation of H_2_ as a bonus byproduct). Carbon particles provide an attractively cheap and readily available support for applications of redox enzymes. Whether the approaches we describe here can be extended beyond the arena of fine chemicals into the larger-scale production of commodity chemicals or biofuels will depend on how readily the metalloenzymes described in this review can be scaled for cheap, high-yield production, and this area remains largely unexplored.

## Abbreviations

AA: ascorbic acid
ADH: alcohol dehydrogenase
ATR: attenuated total reflectance
BP2000: Black Pearls 2000
CODH: carbon monoxide dehydrogenase
*ee*: enantiomeric excess
Hyd1: *E. coli* hydrogenase 1
Hyd2: *E. coli* hydrogenase 2
IR: infrared
ITO: indium tin oxide
MBH: membrane-bound hydrogenase
NAD(P)^+^: nicotinamide adenine dinucleotide (phosphate) oxidised
NAD(P)H: nicotinamide adenine dinucleotide (phosphate) reduced
*t*_1/2_: half-life
TEOA: triethanolamine
TOF: turnover frequency
TTN: total turnover number; TN, turnover number.
